# Post-Pandemic Evolution of Suicide Risk in Children and Adolescents Attending a General Hospital Accident and Emergency Department

**DOI:** 10.3390/healthcare12100977

**Published:** 2024-05-09

**Authors:** Ana Maciá-Casas, Javier de la Iglesia-Larrad, Llanyra García-Ullán, Belén Refoyo-Matellán, Clara Munaiz-Cossío, Sara Díaz-Trejo, Vanesa Berdión-Marcos, Julia Calama-Martín, Carlos Roncero, Jesús Pérez

**Affiliations:** 1 Psychiatry Service, Salamanca Healthcare Complex (CAUSA), 37007 Salamanca, Spain; amacia@saludcastillayleon.es (A.M.-C.); jignacioiglesia@saludcastillayleon.es (J.d.l.I.-L.); mlullan@saludcastillayleon.es (L.G.-U.); brefoyo@saludcastillayleon.es (B.R.-M.); cmunaiz@saludcastillayleon.es (C.M.-C.); sdiaztr@saludcastillayleon.es (S.D.-T.); vberdion@saludcastillayleon.es (V.B.-M.); jcalama@saludcastillayleon.es (J.C.-M.); carlosroncero@usal.es (C.R.); 2 Neuroscience Division, Institute of Biomedical Research of Salamanca (IBSAL), 37007 Salamanca, Spain; 3 Department of Psychiatry, Faculty of Medicine, University of Salamanca, 37001 Salamanca, Spain; 4 Department of Psychiatry, University of Cambridge, Cambridge CB2 3EG, UK; 5 Norwich Medical School, University of East Anglia, Norwich NR4 7UQ, UK

**Keywords:** suicide, suicide ideation, self-harm, self-injury, children, adolescents, emergency

## Abstract

Background: Lockdowns and other health protective measures, such as social distancing, imposed during the COVID-19 pandemic nurtured unprecedented levels of stress and social isolation around the world. This scenario triggered an increase in suicide thoughts and self-harm behaviours among children and young people. However, the longer-term impact of the pandemic on children’s and adolescents’ mental health, especially with regard to self-harm, is still to be fully discovered. Methods: We carried out a retrospective study where we collected data related to suicide ideation and self-harm behaviours in all patients aged under 18 that required on-call psychiatric services at the General Hospital Accident and Emergency (A&E) department in Salamanca, Spain, during 2019 (pre-pandemic) and in both 2021 and 2022 to capture possible variation at different time points during the post-pandemic period. Results: A total of 316 patients aged under 18 were seen by on-call psychiatric services at the A&E department during the three time periods: 78 in 2019, 98 in 2021 and 140 in 2022. The mean age was 15.12 (SD 2.25) and females represented more than twice the number of males each year. More than half of all patients assessed during 2022 disclosed suicide thoughts, whilst in 2019, it was near 25%. This increase in suicide ideation rates was more marked among females (X^2^ = 15.127; *p* = 0.001), those aged over 15 (X^2^ = 16.437; *p* < 0.001) and/or those with a previous history of mental health problems (X^2^ = 17.823; *p* < 0.001). We identified an increase in the proportion of males with suicide ideas, especially between 2021 and 2022 (X^2^ = 8.396; *p* = 0.015). Conclusions: Our study suggests that children’s and adolescents’ demand for urgent mental healthcare and their clinical presentations in A&E departments with suicide thoughts and/or self-injuries do not seem to be declining after the pandemic but increasing over time. More research is warranted to understand possible factors involved in this sustained upward trend.

## 1. Introduction

On 11 March 2020, the World Health Organization (WHO) declared the COVID-19 pandemic [1], which required an urgent, unprecedented adaptation of healthcare systems [2]. Lockdowns and other measures, such as social distancing, were imposed around the world to stop the spread of the disease-causing virus SARS-CoV-2, save the lives of those that were considered more vulnerable, such as the elderly, and prevent the collapse of healthcare services. Lockdown measures included social distancing and ‘stay-at-home’ and ‘work-from-home’ policies [3] that nurtured unprecedented levels of social isolation. This scenario gradually raised psychological distress, or worsened previous mental health problems, in our communities, especially among children and young people [4], which eventually triggered suicide thoughts and self-harm behaviours that required urgent attention by medical emergency services [5].

Studies worldwide found an overall increase in the prevalence of suicidal ideation and attempts and in the rate of death by suicide during the pandemic period [5]. For instance, in 2020, at the climax of the COVID-19 pandemic, 3941 people committed suicide in Spain. This represented a 7.4% increase from the previous year and was the highest ever reported annual figure since the Spanish collection of suicide data began in 1906 [6,7]. A total of 14 suicides out of the 3941 were attributed to children aged under 15, doubling the cases reported in 2019 for that age group [7]. In 2021, suicides of people under 18 increased by 57% compared to 2020, reaching a total of 22 cases [8]. However, despite being the first non-disease-related cause of death in people under 30, Spain still does not have a national strategy for suicide prevention [7].

Whilst the immediate impact of the pandemic on children’s and adolescents’ suicide rates and acts has been acknowledged and widely reported, little is known about the prevalence of acute self-harm ideation and attempts that may precede suicide when the pandemic began to tail off and lockdown measures were relaxed (mostly late 2020 and 2021) or, more recently, at the beginning of what could be called the post-pandemic era (2022). Indeed, the reported effects of the pandemic and associated confinements on suicidality in acute, hospital settings may have been biased by the imposed movement restrictions or more contained help-seeking behaviours due to fears of becoming infected in healthcare settings and therefore may manifest later or worsen over time [9,10,11].

In this study, we aimed to answer the question of whether suicide risk in children and adolescents has increased following the COVID-19 pandemic. To explore this, we analysed the post-pandemic trend in suicidality and self-injuries in children and adolescents by comparing the rates of suicide ideation and self-harm behaviours that required psychiatric assessment and/or intervention in an accident and emergency (A&E) department before and at different time points after the most severe pandemic outbreaks in Spain.

## 2. Materials and Methods

### 2.1. Design and Sample

To achieve our aim, we carried out a retrospective, observational study where we collected data related to the presence or absence of acute suicide ideation and self-harm behaviours in all children and adolescents under 18 that required psychiatric assistance at the Salamanca University Hospital (Hospital Universitario de Salamanca (HUS)) A&E department, in Salamanca, Spain, during 2019 (pre-pandemic) and in both 2021 and 2022; this enabled us to capture possible variation at different time points during the post-pandemic period. We did not apply any exclusion criteria.

We did not include data related to 2020 given the difficulties we could encounter in tracking these presentations in healthcare records during such an unprecedented public health crisis that involved urgent reorganisation, both functionally and structurally, of local health services, which were gradually restored to their former functions and settings by the end of 2020 and the beginning of 2021. In addition, attendance to emergency services in 2020 may have been affected by several factors, such as the imposed public movement restrictions or the fear of becoming infected in hospital settings, amongst others.

### 2.2. Setting

As stated above, this study was performed at the Salamanca University Hospital (Hospital Universitario de Salamanca (HUS)) A&E department. This hospital provides healthcare to the population residing in the city of Salamanca and its province [2], situated in northwest Spain [12]. The latest Spanish National Institute of Statistics (Instituto Nacional de Estadística (INE)) census data [13] indicate that this province has a total population of 327,338 (168,464 women and 158,874 men), with 49,995 (24,309 women and 25,646 men) of these being under 18 years old [14].

According to healthcare referral protocols at HUS, any patient attending the A&E department that discloses suicide ideas, commits any form of self-harm or presents with a suspicion of such behaviour, either with suicide intentions or not, must be referred to the psychiatry on-call service for a mental health assessment. This protocol implies that every patient, regardless of their age, who presents to emergency services with suicide ideas and/or signs of self-harm is seen by a clinical psychiatrist.

### 2.3. Study Variables and Data Collection

We collected all information related to suicide thoughts and self-harm behaviours for all patients aged under 18 that attended the HUS A&E department for the three years under study. Following current scientific consensus, suicide ideation referred to thoughts aiming at one’s own death [15], and self-harm behaviours included attempted and completed suicide, but also intentional self-injuries, regardless of the motives behind them [10,16]. We adopted this definition to avoid difficulties discerning suicide attempts or intentions in some self-harm manifestations in children and adolescents, especially in cross-sectional diagnostic evaluations performed in emergency departments.

In addition, we collected basic sociodemographic information for all patients, specifically age and sex, and information related to psychiatric history and mental health service use, such as the number of visits to the A&E department for psychiatric reasons within the previous 12 months, and the level of follow-up that was prescribed by the on call psychiatrist after the assessment, differentiating between ‘non-intensive’ (the standard mental healthcare provided by child and adolescent community mental health services in Salamanca) and ‘intensive’, such as admission to a psychiatric ward or request for urgent follow-up appointments by the community services within a week to monitor risk closely.

All of this information was obtained from the HUS electronic clinical record system, Jimena 4, and exported to an anonymised database purposely designed for this study.

### 2.4. Statistical Analysis

We distributed all of the study variables categorically (bivariate) for clearer reporting, using both absolute and relative frequencies. We used the chi-square test to make comparisons for the different study variables and considered that there was statistical significance if the *p*-value was below 0.05. We carried out the analyses with the statistical software package SPSS version 15.

### 2.5. Ethical Considerations

The present study was conducted in accordance with the protocol and principles of the current version of the Declaration of Helsinki. Permission to conduct this study was granted by the Research Ethics Committee at the Salamanca University Healthcare Complex (ref: 2023/09 1422) on 27 September 2023.

## 3. Results

### 3.1. Sociodemographic Characteristics and Mental Health Service Use

A total of 316 patients under 18 years old were seen by psychiatrists on call at the HUS A&E department during the three study periods, 78 in 2019, 98 in 2021 and 140 in 2022, showing a remarkable increase in the demand for urgent psychiatric care over time.

The mean age for the whole sample was 15.12 (SD 2.25), with no difference in the distribution of those under and over 15 across the three years. The female sex was most prevalent, representing more than twice the number of male patients every year. We did not find significant differences in distributions for either age or sex between the three years, or for the presence of psychiatric history and the number of visits to A&E services for other mental health assessments within the previous 12 months. However, the intensity of care prescribed after attendance to the emergency department varied significantly, with a higher proportion of patients receiving ‘intensive’ follow-ups post-pandemic, especially among those seen in 2021 (see Table 1).

### 3.2. Suicide Ideation and Self-Harm Behaviour Rates

**Figure 1:**
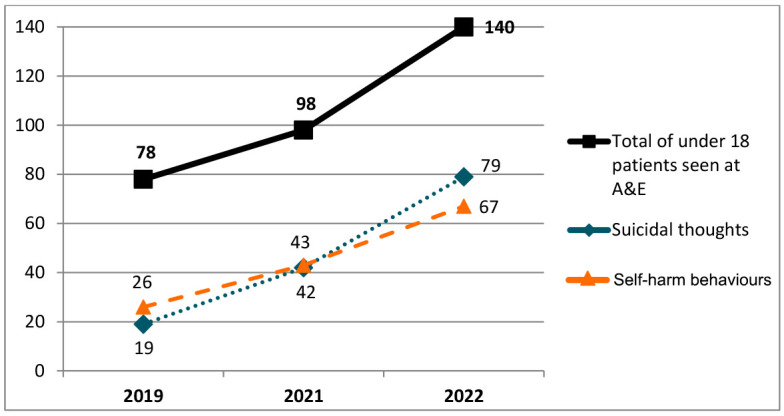
The total number of patients under 18 seen at the HUS A&E department due to mental health problems in 2019, 2021 and 2022, and the number of those patients that disclosed suicide thoughts or self-harm behaviours.

The increase in A&E service demand was also reflected in the increased number of children and adolescents presenting with suicide risk assessed every year in that clinical setting. For instance, in 2022, 60 more patients disclosed suicide ideas than in 2019 (79 vs. 19 patients). The evolution of this service demand is graphically represented in Figure 1.

The proportion of patients with suicide ideation within each year increased significantly over time; for example, more than half of those under 18 assessed in the A&E department during 2022 expressed acute suicide thoughts, whilst in 2019, this number was only around 25%. Interestingly, the significant rise in the percentage of patients with suicide ideation per year was not mirrored in a statistically significant increase in self-harm behaviours. Nonetheless, almost 48% of the patients seen in A&E in 2022 had self-harmed versus 33% three years earlier (see Table 1).

To date, there has not been suicides reported for any of the patients included in this study.

### 3.3. Distribution of Suicide Ideation and Self-Harm Behaviour Rates Depending on Sociodemographic Characteristics and Mental Health Service Use

The proportion of adolescents aged over 15 with suicide thoughts each year showed a statistically significant increase in 2021 and 2022, reaching 62.4% of all patients seen in 2022. This increase in rates was also found in patients under15 but, in that case, with no statistical significance.

With regard to sex, both male and female patients suffered a significant post-pandemic increase in the proportion of those interviewed in the A&E department with suicide thoughts. Notably, we found a sharp increase in the percentage of male patients with suicide ideas between 2021 and 2022. Also, during the post-pandemic period, especially in 2022, we identified a higher proportion of patients with previous psychiatric history that disclosed lethal ideas. The same pattern emerged in this group regarding self-harm behaviours. Also, despite not being statistically significant, patients that attended A&E post-pandemic who disclosed self-harm ideation were more likely to have previously visited such department due to mental health problems (27% (2019) vs. 43% (2021) and 36% (2022)), suggesting that some of those previous attendances could have been preludes for self-harm crises. The same pattern was found for the prescription of ‘intensive’ vs. ‘non-intensive’ follow-up after the A&E assessment, with the former being slightly more frequent in the patients interviewed during 2021 (48%) and returning to pre-pandemic rates (34%) in 2022 (See Table 2).

## 4. Discussion

The burden of the COVID-19 pandemic on the mental health of children and adolescents and the involvement of mental health professionals working in acute psychiatric care settings has already been widely reported. For instance, Bortoletto et al. [17] found a significant increase in the number of children admitted to acute psychiatric wards due to suicide ideation after the lockdown in Verona (Italy). In Zurich (Switzerland), Berger et al. [18] detected an increase in suicide thoughts in children and adolescents that attended emergency departments. Sara et al. [19] also identified an increase in suicide thoughts among children and adolescents living in New Gales (Australia), although they had already noticed a gradual increase in these symptoms amongst children over the last decade, which was more marked following the pandemic. Sivertsen et al. [20] also detected a surge in mental health problems, including suicide thoughts, across Norwegian youngsters, especially among female patients. This sex association was also found by Du et al. [21], who reported an increase in self-harm behaviours in adolescent just after COVID-19 pandemic lockdowns in China. This group also highlighted single-parent families, loneliness and excessive mobile phone use as contributing factors to self-injuries in adolescents.

In Spain, immediately after the states of emergency declared by the Spanish government, Llorca-Bofí et al. [22] started to observe an increase, still non-statistically significant, in suicide ideation among the children and adolescents residing in Lleida. Fernández et al. [11] also reported a gradual increase in attendances to A&E departments due to suicide ideation of children and adolescents living in the eastern province of Alicante during the 2018–2021 period, which, as in other countries, was mostly associated with the female sex. In addition, Gracia-Liso et al. [23], in the northeastern region of Catalonia, noticed a decrease in the age of presentation to psychiatric emergency services with suicide ideation. Gracia et al. [24] found, in the same region, an increase in suicide attempts among adolescents compared to pre-pandemic times, which was, again, predominantly associated with the female sex. This evidence, also found in other countries as stated before, indicates that female adolescents with mental health problems may require more careful assessment and mental healthcare planning to avoid an increase in suicide risk over time.

Most of these studies analysing suicide activity in children and adolescents during the pandemic have mainly focused on the lockdown periods (2020) or soon after (2020 and 2021). Whilst this information is completely relevant, the effect of confinements on suicide thoughts and self-harm behaviours reported from hospital sources might have still been affected by a decrease in visits to the emergency department during the initial pandemic outbreaks, mainly due to movement restrictions, uncertainty surrounding this unparalleled health crisis or, simply, fears of becoming infected in healthcare settings. In our study, we aimed to overcome this limitation by looking into the effect of the pandemic on the distribution of self-harm ideation and behaviour rates in children and adolescents evaluated in an A&E department in 2021, but also in 2022. In comparison with 2019 (pre-pandemic), during 2021, when the pandemic gradually faded away, and 2022, the first year that could be considered post-pandemic, our study identified a remarkable upward trend in the number of children and adolescents seen by psychiatric emergency services in the city and province of Salamanca, Spain, that does not seem to be tempered by the passage of time. In parallel, we found an increase in children’s and adolescents’ attendance to A&E expressing suicide ideation or having committed self-harm. Notably, the groups that showed a higher proportional increase in urgent mental healthcare attention for suicide ideation or self-injuries were adolescents over 15 and patients with a previous history of mental health problems. Curiously, although as previously reported, the female sex was much more likely to be seen in A&E for psychiatric reasons, including those related to self-harm, in male patients, we identified a steep surge in suicide thoughts between 2021 and 2022. In general, the level of care required after A&E assessment during the post-pandemic period was more intensive compared to pre-pandemic, suggesting more severe psychiatric morbidity.

Therefore, we have learnt that the increase in suicide ideation and behaviours in children and adolescents in Salamanca has not been limited to the first year post-pandemic. On the contrary, these clinical presentations are still increasing, which should be a public health matter of concern. Further research and a profound analysis of the possible reasons behind what seems to indicate a long-term, and rising, negative evolution of the mental health and emotional wellbeing of our younger populations following the COVID-19 pandemic are warranted to develop and implement evidence-based suicide and self-harm prevention strategies, specifically in Spain, to overturn this worrying trend. To date, we know that possible causes related to the negative impact of the pandemic on the emotional wellbeing of children and the adolescent population include the time that they spend alone, a lack of physical exercise, altered sleep patterns and the much greater use of videogames and social media [25]. In addition, the closure of schools could have harmed students’ mental health, as these are not only teaching centres, but also providers of social interactions with other peers or adults other than immediate family members, providers of physical exercise and regular meals, and monitors of child abuse or neglect [26]. Nevertheless, the long-term negative impact that we are witnessing may be related to a sum of these and other factors, in addition to behavioural patterns that already existed or were brewing before the pandemic, which could have been accelerated and/or sharpened by the irruption of SARS-CoV-2 and its impact on a critical period of physical and social development as a human being [9,10,27].

It should be noted that this study is limited by the fact that our retrospective sample only included evaluations carried out by on-call psychiatric services in the HUS A&E department, not patients seen exclusively by HUS general emergency staff, which is assumed, as stated before, to be very infrequent at HUS given the existing A&E protocols for patients disclosing suicide ideation or self-injury. Also, it did not include those assessed urgently by community mental health services outside of the acute care system. In addition, although in 2021, healthcare services returned to pre-pandemic organisational structures and clinical duties, our study did not evaluate potential societal changes beyond the effect of the COVID-19 pandemic that could have influenced variations in the accessibility of young populations to such services. Furthermore, the sociodemographic characteristics that we identified as having a significant impact on the results, such as the female sex, might have required a much deeper analysis that we could not perform due to the retrospective nature of our study design. Finally, despite this being a real-world study, it is restricted to one site, the city and province of Salamanca, and therefore, our study may need to be replicated elsewhere and our findings compared with other regions nationally and internationally to rule out differences that could be attributed to sociocultural contexts and/or healthcare systems.

## 5. Conclusions

Our study suggests that children’s and adolescents’ demand for psychiatric urgent care and their clinical presentations at A&E departments with suicide thoughts and/or self-injuries do not seem to be declining after the pandemic but increasing over time. Suicide risk behaviours in this population tripled by December 2022 from pre-pandemic times and may still be rising.

More research is warranted to understand the possible factors involved in this sustained upward trend, which may be related to or worsened by the long-term pandemic impact on our younger populations. This understanding should help us develop initiatives and adjustments in healthcare systems to prevent the further deterioration of more children’s and young people’s mental health.

## Figures and Tables

**Table 1:** Sociodemographic and clinical characteristics of patients under 18 seen at HUS A&E department due to mental health problems in 2019, 2021 and 2022.

Categories			2019 (N = 78)	2021 (N = 98)	2022 (N = 140)	Statistical Analysis
Age Median: 15.38 Mean: 15.12 (SD 2.25)	Under 15 years N (%)		31 (39.7%)	42 (42.9%)	55 (39.3%)	X^2^ = 0.330 *p* = 0.848
	Over 15 years N (%)		47 (60.3%)	56 (57.1%)	85 (60.7%)	
Sex	Female N (%)		53 (67.9%)	67 (68.4%)	100 (71.4%)	X^2^ = 0.392 *p* = 0.822
	Male N (%)		25 (32.1%)	31 (31.6%)	40 (28.6%)	
Previous psychiatric history N (%)			51 (65.4%)	68 (69.4%)	110 (78.6%)	X^2^ = 5.042 *p* = 0.080
Number of psychiatric emergency visits		One visit N (%)	60 (76.9%)	69 (70.4%)	98 (70%)	X^2^ = 1.330 *p* = 0.514
		More than one visit N (%)	18 (23.1%)	29 (29.6%)	42 (30%)	
Type of management		Non-intensive N (%)	64 (82.1%)	64 (65.3%)	102 (72.9%)	X^2^ = 6.149 *p* = 0.046
		Intensive N (%)	14 (17.9%)	34 (34.7%)	38 (27.1%)	
Suicide thoughts N (%)			19 (24.4%)	42 (42.9%)	79 (56.4%)	X^2^ = 20.998 *p* < 0.001
Self-harm behaviours N (%)			26 (33.3%)	43 (43.9%)	67 (47.9%)	X^2^ = 4.351 *p* = 0.114

X^2^: chi-square significance test; *p*: *p*-value.

**Table 2:** Prevalence of suicide thoughts and self-harm behaviour in patients aged under 18 seen at HUS A&E department according to age, sex and PPH in 2019, 2021 and 2022.

Suicide Thoughts and Self-Harm Behaviours		2019 (N = 78)	2021 (N = 98)	2022 (N = 140)	Statistical Analysis
Age		<15 years N = 31	<15 years N = 42	<15 years N = 55	
	Suicide thoughts N (%)	7 (22.6%)	15 (35.7%)	26 (47.3%)	X^2^ = 5.242 *p* = 0.073
	Self-harm behaviours N (%)	11 (35.5%)	16 (38.1%)	22 (40%)	X^2^ = 0.172 *p* = 0.918
		>15 years N = 47	>15 years N = 56	>15 years N = 85	
	Suicide thoughts N (%)	12 (25.5%)	27 (48.2%)	53 (62.4%)	X^2^ = 16.437 *p* < 0.001
	Self-harm behaviours N (%)	15 (31.9%)	27 (48.2%)	45 (52.9%)	X^2^ = 5.502 *p* = 0.064
Sex		Female N = 53	Female N = 67	Female N = 100	
	Suicide thoughts N (%)	15 (28.3%)	36 (53.7%)	61 (61%)	X^2^ = 15.127 *p* = 0.001
	Self-harm behaviours N (%)	22 (41.5%)	36 (53.7%)	56 (56%)	X^2^ = 3.055 *p* = 0.217
		Male N = 25	Male N = 31	Male N = 40	
	Suicide thoughts N (%)	4 (16%)	6 (19.4%)	18 (45%)	X^2^ = 8.396 *p* = 0.015
	Self-harm behaviours N (%)	4 (16%)	7 (22.6%)	11 (27.5%)	X^2^ = 1.155 *p* = 0.561
PPH		With PPH N = 51	With PPH N = 68	With PPH N = 110	
	Suicide thoughts N (%)	13 (25.5%)	31 (45.6%)	67 (60.9%)	X^2^ = 17.823 *p* < 0.001
	Self-harm behaviours N (%)	15 (29.4%)	29 (42.6%)	60 (54.5%)	X^2^ = 9.178 *p* = 0.010
		Without PPH N = 27	Without PPH N = 30	Without PPH N = 30	
	Suicide thoughts N (%)	6 (22.2%)	11 (36.7%)	12 (40%)	X^2^ = 2.250 *p* = 0.325
	Self-harm behaviours N (%)	11 (40.7%)	14 (46.7%)	7 (23.3%)	X^2^ = 3.776 *p* = 0.151

X^2^: chi-square significance test; *p*: *p*-value; PPH: previous psychiatric history.

## Data Availability

The data are contained within the article.
